# Nicotinamide Mononucleotide, an Intermediate of NAD^+^ Synthesis, Protects the Heart from Ischemia and Reperfusion

**DOI:** 10.1371/journal.pone.0098972

**Published:** 2014-06-06

**Authors:** Takanobu Yamamoto, Jaemin Byun, Peiyong Zhai, Yoshiyuki Ikeda, Shinichi Oka, Junichi Sadoshima

**Affiliations:** Department of Cell Biology and Molecular Medicine, Cardiovascular Research Institute, Rutgers New Jersey Medical School, Newark, New Jersey, United States of America; Tokai University, Japan

## Abstract

Nicotinamide phosphoribosyltransferase (Nampt), the rate-limiting enzyme for nicotinamide adenine dinucleotide (NAD^+^) synthesis, and Sirt1, an NAD^+^-dependent histone deacetylase, protect the heart against ischemia/reperfusion (I/R). It remains unknown whether Nampt mediates the protective effect of ischemic preconditioning (IPC), whether nicotinamide mononucleotide (NMN, 500 mg/kg), a product of Nampt in the NAD^+^ salvage pathway, mimics the effect of IPC, or whether caloric restriction (CR) upregulates Nampt and protects the heart through a Sirt1-dependent mechanism. IPC upregulated Nampt protein, and the protective effect of IPC against ischemia (30 minutes) and reperfusion (24 hours) was attenuated at both early and late phases in Nampt +/− mice, suggesting that Nampt plays an essential role in mediating the protective effect of IPC. In order to mimic the effect of Nampt, NMN was administered by intraperitoneal injection. NMN significantly increased the level of NAD^+^ in the heart at baseline and prevented a decrease in NAD^+^ during ischemia. NMN protected the heart from I/R injury when it was applied once 30 minutes before ischemia or 4 times just before and during reperfusion, suggesting that exogenous NMN protects the heart from I/R injury in both ischemic and reperfusion phases. The protective effect of NMN was accompanied by decreases in acetylation of FoxO1, but it was not obvious in Sirt1 KO mice, suggesting that the effect of NMN is mediated through activation of Sirt1. Compared to control diet (90% calories), CR (60% calories for 6 weeks) in mice led to a significant reduction in I/R injury, accompanied by upregulation of Nampt. The protective effect of CR against I/R injury was not significant in cardiac-specific Sirt1 KO mice, suggesting that the protective effect of CR is in part mediated through the Nampt-Sirt1 pathway. In conclusion, exogenous application of NMN and CR protects the heart by both mimicking IPC and activating Sirt1.

## Introduction

Ischemia followed by reperfusion (I/R) in the heart is a life-threatening event, and myocardial damage induced by I/R is an important cause of heart failure in patients [1]. At the cellular level, shortly after exposure to ischemia or hypoxia, adenosine triphosphate (ATP) contents rapidly decrease in cardiomyocytes. In order to survive during ischemia or hypoxia, cardiomyocytes switch their metabolism from an aerobic mechanism to an anaerobic one, namely, from fatty acid oxidation to glycolysis [2], and activate autophagy, a self-eating mechanism, to salvage ATP [3]. If ischemia continues, cytochrome c release from mitochondria triggers apoptosis, while mitochondrial permeability transition pore opening is followed by depletion of ATP, decreases in the intracellular pH, increases in Ca^2+^ overload, and eventual necrosis in cardiomyocytes [4]. Rapid reperfusion provides fuel for ATP production, such as glucose and fatty acids, supplies oxygen, and washes out noxious substances derived from necrotic cells, but the rapid recovery of extracellular pH and the oxygen supply results in further Ca^2+^ overload and production of reactive oxygen species (ROS), causing reperfusion injury [5]. Surviving cardiomyocytes suffer from endoplasmic reticulum (ER) stress or oxidative stress, both of which cause cellular malfunctions. Thus, I/R is a series of events that induce nearly unavoidable cell death and serious malfunction in cardiomyocytes, and despite intensive investigations in the field, interventions with clinically proven efficacy remain to be developed.

Ischemic preconditioning (IPC) is a powerful endogenous mechanism of protection against myocardial I/R activated by repetitive brief periods of ischemia and reperfusion. The protection afforded by IPC is mediated through activation of multiple signaling pathways, some of which have been well defined [6]. For example, IPC activates Sirt1, a class III histone deacetylase, and induces deacetylation of lysine residues in proteins, including p53. Inhibition of endogenous Sirt1 with splitmicin, a chemical inhibitor of Sirt1, reverses the protection by IPC, suggesting that Sirt1 mediates the cardioprotective effect of IPC [7]. We have shown previously that downregulation of Sirt1 promotes I/R injury [8], whereas modest upregulation of Sirt1 is sufficient to protect the heart from oxidative stress [9] and I/R injury through deacetylation of FoxO transcription factors [8]. Although these results clearly suggest that stimulation of Sirt1 effectively attenuates the myocardial injury caused by I/R, it remains unclear how Sirt1 is stimulated without relying on interventions to deliver proteins or nucleic acids encoding Sirt1[9]. Since the deacetylase activity of Sirt1 critically depends upon the intracellular level of nicotinamide adenine dinucleotide (NAD^+^) [10], NAD^+^ may be an alternative target for promoting IPC through Sirt1.

In mammalian cells, NAD^+^ is synthesized by four different pathways, including *de novo* synthesis from tryptophan, synthesis from either nicotinamide or nicotinic acid, or conversion of nicotinamide riboside [11]. We have shown previously that nicotinamide phosphoribosyltransferase (Nampt), an enzyme in the mammalian NAD^+^ salvage pathway, plays an important role in mediating NAD^+^ synthesis in cardiomyocytes [12]. Nampt catalyzes the transfer of a phosphoribosyl residue from phosphoribosyl pyrophosphate (PRPP) to nicotinamide to produce nicotinamide mononucleotide (NMN), which is in turn converted to NAD^+^ through NMN adenylyltransferase. Endogenous Nampt is downregulated in response to I/R, and cardiac-specific overexpression of Nampt protects the heart from both ischemia and I/R [13], offering proof of the concept that stimulating Nampt can reduce I/R injury. Interestingly, NMN, the product of the rate-limiting enzyme in the NAD^+^ salvage pathway, has been used previously in order to achieve the same effect as overexpression of Nampt, where the administration of NMN caused a partial recovery of insulin secretion from pancreatic β-cells [14] in Nampt +/− mice.

We here hypothesized that NMN can be used to compensate for the downregulation of Nampt and protect the heart during I/R. The goals in this study were 1) to elucidate whether Nampt mediates the protective effect of preconditioning against I/R using Nampt +/− mice, and if so, 2) to test whether exogenous application of NMN mimics the effect of preconditioning, and 3) to test whether caloric restriction (CR) upregulates Nampt and protects the heart from I/R through a Sirt1-dependent mechanism.

## Materials and Methods

### Genetically Altered Mouse Models

Systemic Nampt heterozygous knockout (Nampt +/−) mice were provided by Dr. S. Yamanaka (Kyoto University, Japan). Cardiac-specific Sirt1 knockout (Sirt1KO) mice were generated by crossing Sirt1*flox/flox* mice (Jackson Laboratory) with C57BL/6J background and α-myosin heavy chain promoter–driven Cre mice (αMHC-Cre, courtesy of Dr. M. Schneider, Imperial College, London, UK) as described previously [13]. All Sirt1*flox/flox* (control) and Sirt1*flox/flox*, α*MHC-Cre* (Sirt1KO) mice were backcrossed to C57BL/6J background. Transgenic mice with cardiac-specific expression of mRFP-GFP-LC3 have been described [15]. All animal protocols were approved by the Institutional Animal Care and Use Committee of Rutgers New Jersey Medical School.

### Echocardiography

Echocardiography was performed as described, using ultrasonography (Acuson Sequoia C256; Siemens Medical Solutions).

### Quantitative RT-PCR

Relative mRNA levels were examined by quantitative RT-PCR. Total RNA was prepared from the left ventricle using Trizol (Invitrogen). Then, cDNA was generated using 2 µg total RNA and M-MLV Reverse transcriptase (Promega). Using Maxima SYBR Green qPCR master mix (Fermentas), real-time RT-PCR was performed under the following conditions: 94°C for 15 min; 40 cycles of 94°C for 5 s; 57°C for 10 s; 72°C for 10 s, and a final elongation at 72°C for 15 min. The mean value of control mice was expressed as 1. PCRs were carried out using the following oligonucleotide primers:

18S rRNA: Fw: GTA ACC CGT TGA ACC CCA TT, Rv: CCA TCC AAT CGG TAG TAG CG

Nampt: Fw: TCC TGC TGG CCA CCG ACT CG, Rv: ACT TTG CTT GTG TTG GGT GGG T

### Antibodies

The antibodies used in this study include Nampt antibody (abcam, Cambridge, MA), FoxO1 antibody (Santa Cruz Biotechnology Inc, Santa Cruz, CA), GAPDH antibody (Sigma-Aldrich, St. Louis, MO), thioredoxin1 antibody (generated by this laboratory), Ac-FoxO1 antibody, Akt antibody, phospho-specific Akt antibody, ERK1/2 antibody, phospho-specific ERK1/2 antibody, STAT3 antibody, phospho-specific STAT3 antibody, Bcl2 antibody, Bcl-xL antibody, Bax antibody (Cell Signaling Technology, Danvers, MA), and MnSOD antibody (BD Transduction Laboratory, San Jose, CA). Ly.6B.2 antibody (AbD Serotec, Raleigh, NC)

### Ischemia and I/R In Vivo

Mice were housed in a temperature-controlled environment with 12-hour light/dark cycles where they received food and water *ad libitum*. Mice were anesthetized by intraperitoneal injection of pentobarbital sodium (50 mg/kg). A rodent ventilator (model 683, Harvard Apparatus Inc) was used with 65% oxygen. The animals were kept warm with heat lamps. Rectal temperature was monitored and maintained between 36°C and 37°C. The chest was opened by a horizontal incision at the third intercostal space. I/R was achieved by ligating the anterior descending branch of the left coronary artery with an 8-0 Prolene suture, with silicon tubing (1 mm outer diameter) placed on top of the left anterior descending coronary artery, 2 mm below the border between the left atrium and left ventricle (LV). Ischemia was confirmed by ECG change (ST elevation). After occlusion for 30 minutes, the silicon tubing was removed to achieve reperfusion, and the rib space and overlying muscles were closed. When recovered from anesthesia, the mice were extubated and returned to their cages. They were housed in a climate-controlled environment. Twenty-four hours after reperfusion, the animals were reanesthetized and intubated, and the chest was opened. After the heart was arrested at the diastolic phase by KCl injection, the ascending aorta was cannulated and perfused with saline to wash out blood. The left anterior descending coronary artery was occluded with the same suture, which had been left at the site of the ligation. To demarcate the ischemic area at risk (AAR), Alcian blue dye (1%) was perfused into the aorta and coronary arteries. Hearts were excised, and LVs were sliced into 1-mm-thick cross sections. The heart sections were then incubated with a 1% triphenyltetrazolium chloride solution at 37°C for 15 minutes. The infarct area (pale), the AAR (not blue), and the total LV area from both sides of each section were measured with the use of Adobe Photoshop (Adobe Systems Inc), and the values obtained were averaged. The percentages of area of infarction and AAR of each section were multiplied by the weight of the section and then totaled from all sections. AAR/LV and infarct area/AAR were expressed as percentages.

### NAD^+^ Measurements

NAD^+^ was measured, using the EnzyChrom NAD^+^/NADH Assay Kit according to the protocol of the manufacturer (ECND-100, Bioassay Systems, Hayward, California). Hearts were dissected from mice and weighed. The hearts were homogenized with 100 µL NAD^+^ extraction buffer per 5 mg tissue with a handheld tissue homogenizer. Extracts were heated for five minutes at 60°C and 20 µL of assay buffer was added, followed by 100 µL NADH extraction buffer (to neutralize the extracts). Mixtures were vortexed and centrifuged at 12,000 g for five minutes. Supernatants (20 µL) were then mixed with 40 µL Working Reagent containing 30 µL assay buffer, 0.5 µL enzyme A, 0.5 µL enzyme B, 7 µL lactate and 7 µL tetrazolium dye. Optical density at 565 nm was recorded at time zero (OD0) and at 15 minutes (OD15) using a 96-well plate reader spectrophotometer. The difference in absorbance between time zero and 15 minutes (ΔOD) of the sample was compared with that of the standard solutions to determine the NAD^+^ concentration. For measurement of NADH, the hearts were extracted with NADH extraction buffer and neutralized with NAD^+^ extraction buffer. The rest of the procedure was the same as for NAD^+^ measurement. In order to avoid variability due to the circadian regulation of the level of Nampt and/or NAD^+^, all experiments, both *in vivo* and *in vitro*, were conducted during the day time.

### Caloric Restriction

Eight- to 12-week-old male mice were randomly divided into 2 groups. All mice were fed *ad libitum* for 1 week and their intake was monitored. For the control normal diet (ND) group, the mice were fed 90% of the average intake of the *ad libitum* phase. For the caloric restriction (CR) group, the mice were fed 90% (10% reduction) of the average intake of the *ad libitum* phase for 1 week followed by 60% (40% reduction). For the *ad libitum* phase and 10% reduction diet, Dustless Precision Pellets 1 gm (Bio-Serv #F05312, Frenchtown, New Jersey) were fed. For the 40% reduction diet, Dustless Precision Pellets 1 gm 40% DR (Bio-Serv #F05314) were fed.

### Evaluation of Neutrophil Infiltration

Hearts from mice sacrificed after 24 h of reperfusion were frozen in OCT and cut serially from the occlusion locus to the apex in 10 mm sections. Immunostaining for neutrophils was performed on the cryo-sectioned slide with primary antibody (anti-mouse Ly-6B.2 antibody, AbD Serotec, Raleigh, NC) and Alexa Fluor 488 Dye-conjugated secondary antibody (Invitrogen BD Transduction Laboratory, San Jose, CA). Slides were mounted using a reagent containing 4′,6-diamidino-2-phenylindole (DAPI) (Vectashield, Vector Laboratories Inc, Burlingame, CA). Quantifications were performed with ImageJ (NIH). The results were analyzed with relative ratio of the surface of neutrophil to the total surface of cardiomyocytes.

### ELISA Assays for CXCL1 and CXCL2

After I/R, the blood was taken from the orbital sinus and incubated at room temperature for 30 minutes and then centrifuged at 2,000 rpm for 15 minutes at 4°C. The serum layer was transferred into a new tube and preserved at −80°C until the assay. The serum level of chemokine (C-X-C motif) ligand 1 (CXCL1) and CXCL2 was measured by a colorimetric enzyme-linked immunosorbent assay (ELISA) (R&D Systems, Pittsburgh, PA).

### Evaluation of Fluorescent LC3 Puncta

The method of evaluating tandem fluorescent LC3 puncta using Ad-tf-LC3 has been described previously [16]. For in vivo determination of the number of fluorescent LC3 dots, fresh heart slices were embedded in Tissue-Tek OCT compound (Sakura Finetechnical Co.) and frozen at −80°C. Sections 10 µm thick were obtained from the frozen tissue samples using a cryostat (CM3050 S; Leica), air-dried for 30 min, fixed by washing in 95% ethanol for 10 min, mounted using a reagent containing DAPI, and viewed under a fluorescence microscope (Nikon Eclipse E800). The number of GFP and mRFP dots was determined by manual counting of fluorescent puncta from at least 4 different myocyte preparations with a 60X objective. At least 50 cells were scored in each experiment. The nuclear number was evaluated by counting the number of DAPI-stained nuclei in the same field. The number of dots/cell was obtained by dividing the total number of dots by the number of nuclei in each microscopic field.

### TUNEL Staining

DNA fragmentation was detected in situ with the use of terminal deoxynucleotidyl transferase dUTP nick end labeling (TUNEL), as described. Nuclear density was determined by manual counting of DAPI-stained nuclei in 6 fields for each animal with the 40x objective, and the number of TUNEL-positive nuclei was counted by examining the entire section with the same power objective.

### ATP Assays

Primary cultured myocytes were plated on 12 well dishes with 1 ml/well myocyte medium (BrdU (+)). One day after the isolation, the medium was exchanged for fresh myocyte medium (BrdU(-)) for 2 days. The cells were treated with NMN and lysed with 100 ml/well Somatic Cell ATP releasing reagent (Sigma). ATP content was measured by luminometric assay using an ATP bioluminescent assay kit (Sigma). The ATP assay mix was diluted with the dilution buffer (500 fold dilution). The diluted ATP assay mix (50 ml) was then mixed with10 ml of cell lysate and the luminescence was measured by luminometer for 10 seconds. The ATP content was normalized by protein content.

### Statistics

Statistical analyses between groups were performed by 1-way ANOVA, and differences among group means were evaluated using Fisher's project least significant difference post test procedure for group data, with a probability value less than 0.05 considered significant.

## Results

### Nampt is upregulated by ischemic preconditioning

**Figure 1:**
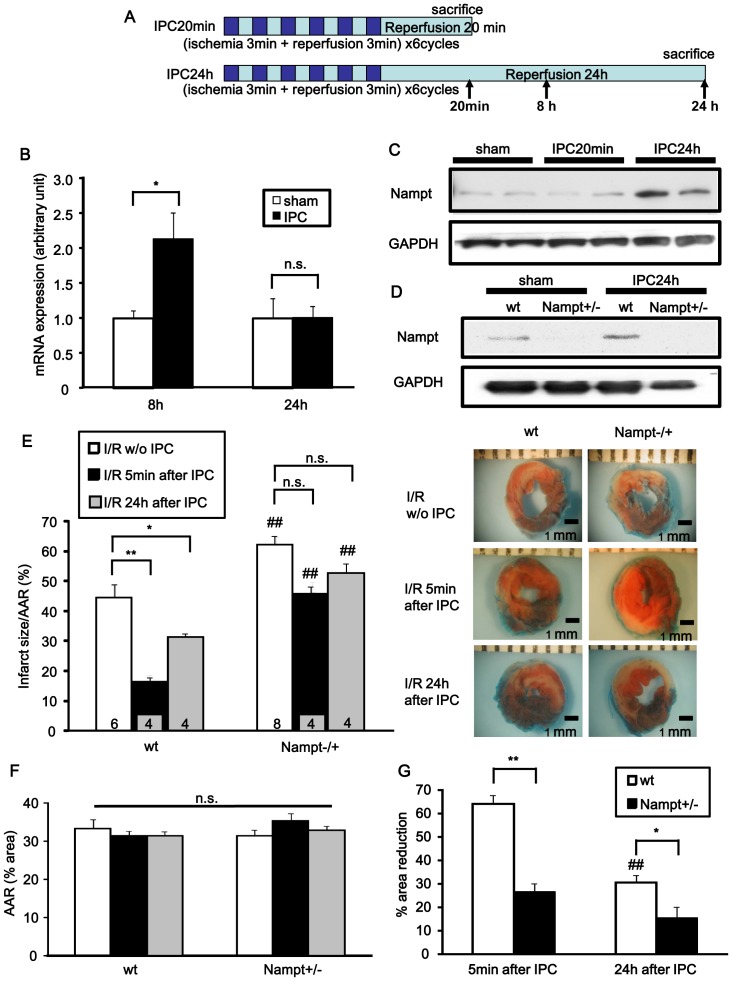
Nampt expression is upregulated by ischemic preconditioning (IPC). A, The IPC protocol for wild-type mice. Mice on C57BL/6 background were subjected to 6 cycles of 3 minutes of ischemia plus 3 minutes of reperfusion. Sham groups were subjected to open chest surgery only. Arrows indicate the timing of biochemical analyses. B, Nampt mRNA expression 8 hours and 24 hours after IPC was determined by quantitative RT-PCR. n = 4. C, Nampt protein expression 20 minutes and 24 hours after IPC was determined by Western blot. D, Nampt protein expression with or without IPC in Nampt +/− mice and their wild-type littermates. E-G, Nampt +/− and littermate wild-type mice were subjected to IPC as shown in A. Five minutes or 24 hours after IPC, the mice were subjected to ischemia (30 minutes)/reperfusion (24 hours) (I/R). Some mice were subjected to I/R without IPC. Infarct size/AAR (E), AAR (F) and % reduction in infarct size compared to those without IPC (G) are shown. In E and F, ## p<0.01 vs. wild-type littermates subjected to the same surgery. n = 4 to 8. In G, ## p<0.01 vs. wild-type littermates subjected to I/R 5 minutes after IPC. In B, E, F and G, n.s., not significant; * p<0.05, ** p<0.01.

We examined whether expression of Nampt, the rate-limiting enzyme for NAD^+^ synthesis in the heart, is regulated by IPC. Mice were subjected to IPC: 6 cycles of 3 min ischemia plus 3 min reperfusion or sham operation (Figure 1A). Nampt mRNA was upregulated 8 hours after IPC, but there was no significant difference between the Nampt mRNA levels of sham and IPC groups 24 hours after IPC, as determined by quantitative RT-PCR (Figure 1B). Nampt protein was upregulated 24 hours after IPC, but there was no significant difference between sham and IPC groups 20 min after IPC (Figure 1C). In order to evaluate the role of Nampt in mediating the cardioprotective effect of IPC, we used systemic Nampt +/− mice (Figure S1 in File S1). At 3 months of age the cardiac phenotype of Nampt+/− mice was not significantly different from that of wild-type mice (Tables S1 and S2 in File S1). We applied ischemia (20 min) followed by 24 hours of reperfusion (I/R) to Nampt+/− and wild type mice either 5 min or 24 hours after IPC. As expected, the level of Nampt protein in the heart was lower in the sham-operated Nampt +/− mice and the upregulation of Nampt observed in wild-type mice 24 hours after IPC was abolished in Nampt +/− mice (Figure 1D). In the wild-type mice subjected to IPC 5 min or 24 hours before I/R, the infarct area was reduced by 63. 9% or 30.4%, respectively, compared to that in mice that received I/R without IPC. In Nampt +/− mice subjected to IPC 5 min or 24 hours before I/R, however, the infarct area was reduced by only 26.3% (p<0.01 vs wild-type mice) or 15.2% (p<0.05 vs wild-type mice), respectively, compared to that in mice that received I/R without IPC (Figure1E-G and Figure S2 in File S1). These results indicate that the cardioprotective effect of IPC against I/R is in part mediated by Nampt.

### NAD^+^ content in the heart is reduced in Nampt +/− mice after ischemia

**Figure 2:**
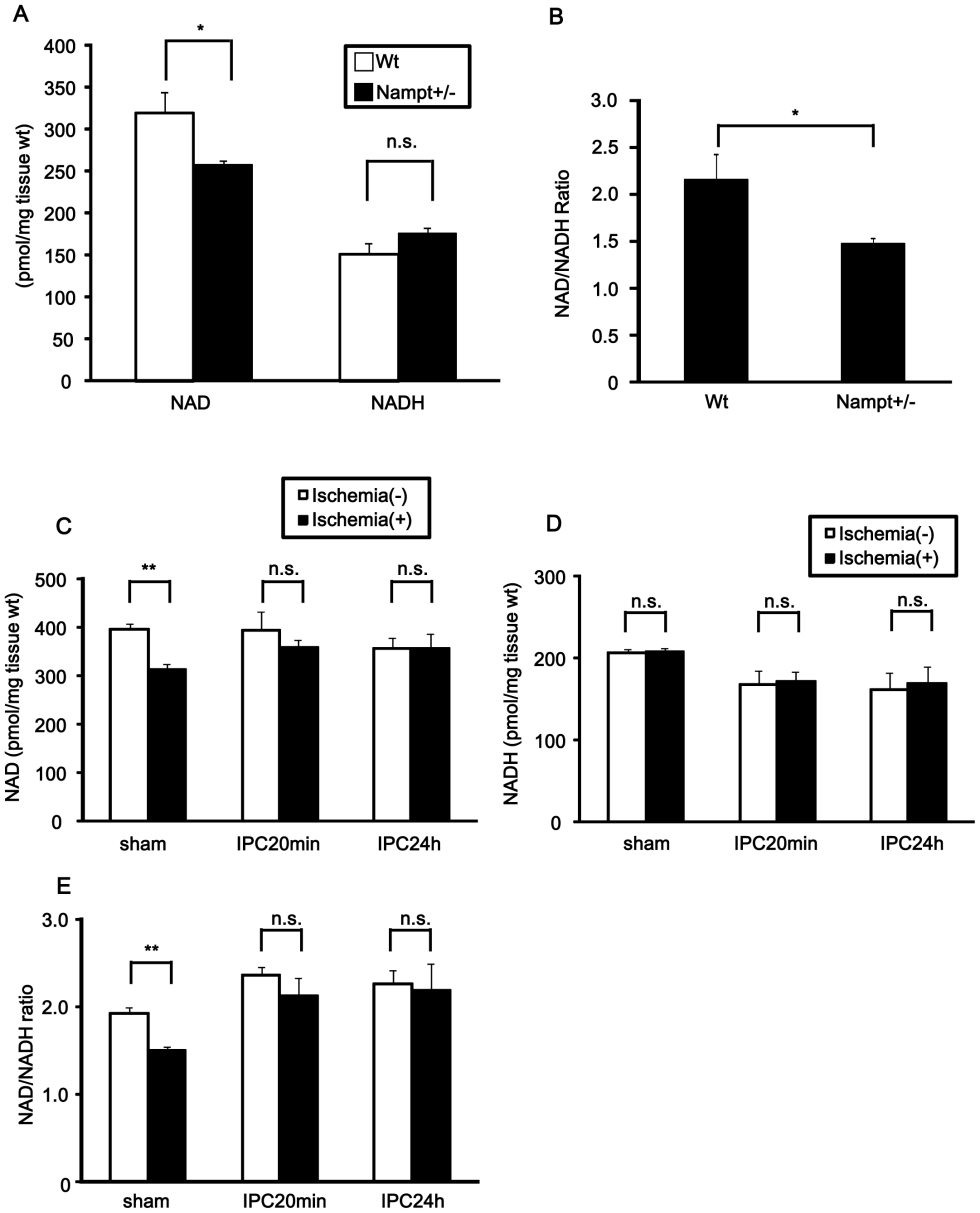
NAD^+^ content in the heart is reduced in Nampt +/− mice. A and B, Heart homogenates were prepared from Nampt +/− mice and their wild-type (Wt) littermates. A, NAD^+^ and NADH contents. B, NAD^+^/NADH ratio. C-E, Mice were subjected to sham procedure or IPC, as shown in Figure 1A. Mice were then subjected to either 30 minutes ischemia or sham operation 5 minutes after the sham procedure or either 5 minutes or 24 hours after IPC. C, NAD^+^ contents. D, NADH contents. E, NAD^+^/NADH ratio. In A-E, n = 4. n.s., not significant; * p<0.05, ** p<0.01.

We evaluated the NAD^+^ and NADH content in the hearts of Nampt +/− mice at baseline (Figure 2A and 2B). Although the NADH content in the hearts of Nampt +/− mice was not significantly different from that in wild-type littermates, the NAD^+^ content and NAD^+^/NADH ratio at baseline were significantly lower in Nampt +/− mice than in wild-type littermates, suggesting that endogenous Nampt plays an essential role in maintaining the NAD^+^ level and NAD^+^/NADH in the mouse heart. We have shown previously that prolonged myocardial ischemia time-dependently downregulates Nampt in the mouse heart [13]. Although the NAD^+^ content and NAD^+^/NADH ratio in the mouse heart after 30 min of ischemia were decreased without IPC, they were preserved when ischemia was applied either 5 min or 24 hours after IPC (Figure S3 in File S1, Figure 2C-E). The NADH content did not change after 30 min of ischemia with or without IPC. These results suggest that IPC acts to maintain the level of NAD^+^ and the NAD^+^/NADH ratio during myocardial ischemia.

### NMN increases the NAD^+^ and NADH content in the heart

**Figure 3:**
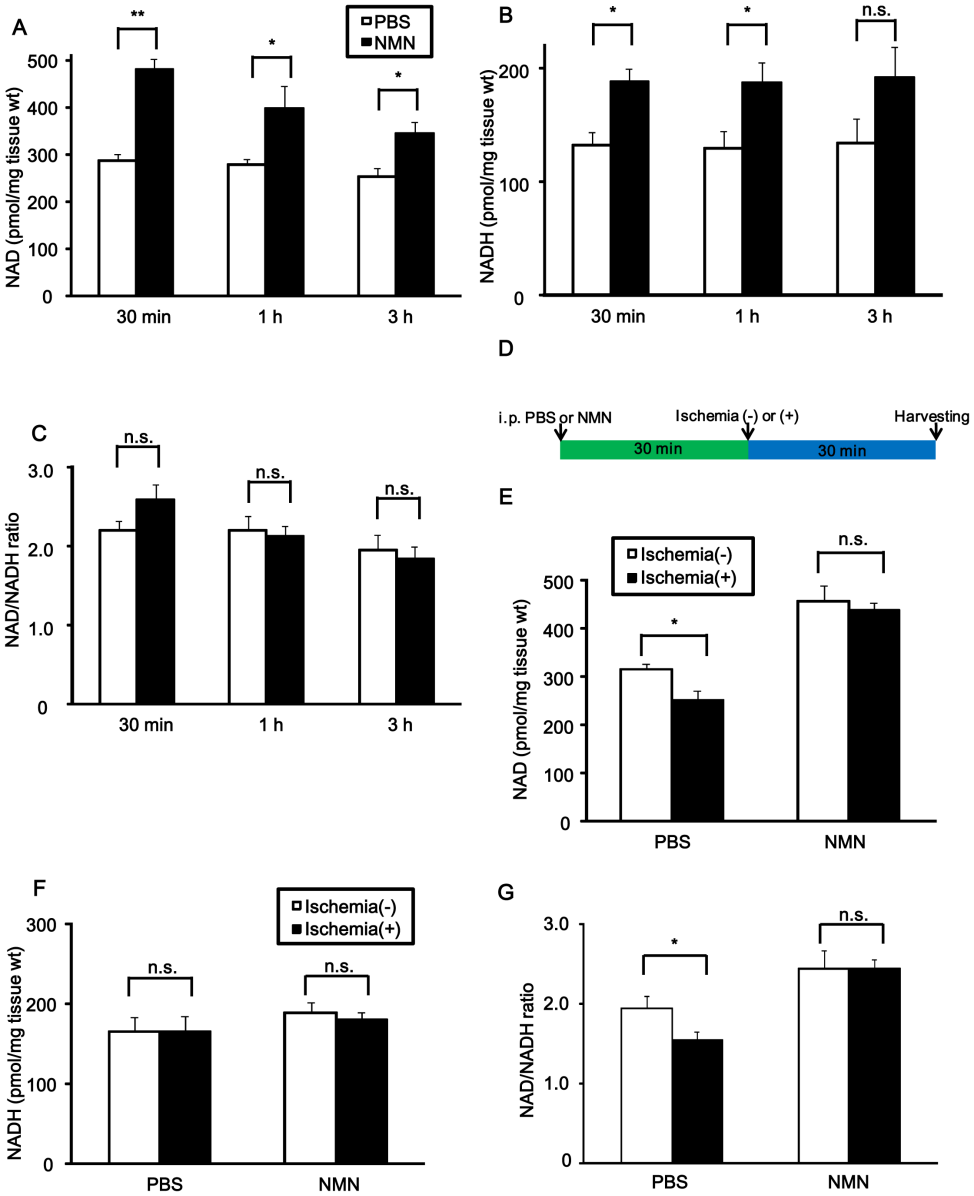
NAD^+^ content after NMN administration. A-C, NMN (500 mg/kg) or vehicle (phosphate buffered saline) was administered to mice and the hearts were harvested at the indicated time points. n = 3 to 4. A, NAD^+^ contents. B, NADH contents. C, NAD^+^/NADH ratio. D, The protocol for NMN injection followed by 30 minutes ischemia for the measurement of NAD^+^, NADH and NAD^+^/NADH in E-G. E, NAD^+^ contents. F, NADH contents. G, NAD^+^/NADH ratio. In E-G, n = 4. In A-C and E-G, n.s., not significant, * p<0.05, ** p<0.01.

Since Nampt is the rate-limiting enzyme for NAD^+^ synthesis [11], NAD^+^ content in the heart may be increased by the administration of NMN, the product of the enzymatic reaction of Nampt with its substrate, nicotinamide. We measured NAD^+^ and NADH contents 30 min, 1 hour and 3 hours after intraperitoneal (i.p.) injection (500 mg/kg body weight) of NMN (Figure 3A and 3B). Both NAD^+^ and NADH contents were increased as early as 30 min after the administration of NMN. The NAD^+^/NADH ratio was not significantly affected by NMN (Figure 3C). Since NMN increases the NAD^+^ content 30 min after injection and the increase persists for more than 1 hour, we tested the effect of NMN upon the ischemia-induced reduction in the NAD^+^ content by injecting it 30 min before ischemia and harvesting the heart after 30 min of ischemia (Figure 3D). As expected, NMN increased both NAD^+^ content and the NAD^+^/NADH ratio in the heart with or without ischemia. Moreover, the decreases in the NAD^+^ content and NAD^+^/NADH ratio observed after 30 min of ischemia in PBS-injected mice were normalized after NMN administration (Figure 3E-G).

### NMN administration reduces the infarct area after I/R

**Figure 4:**
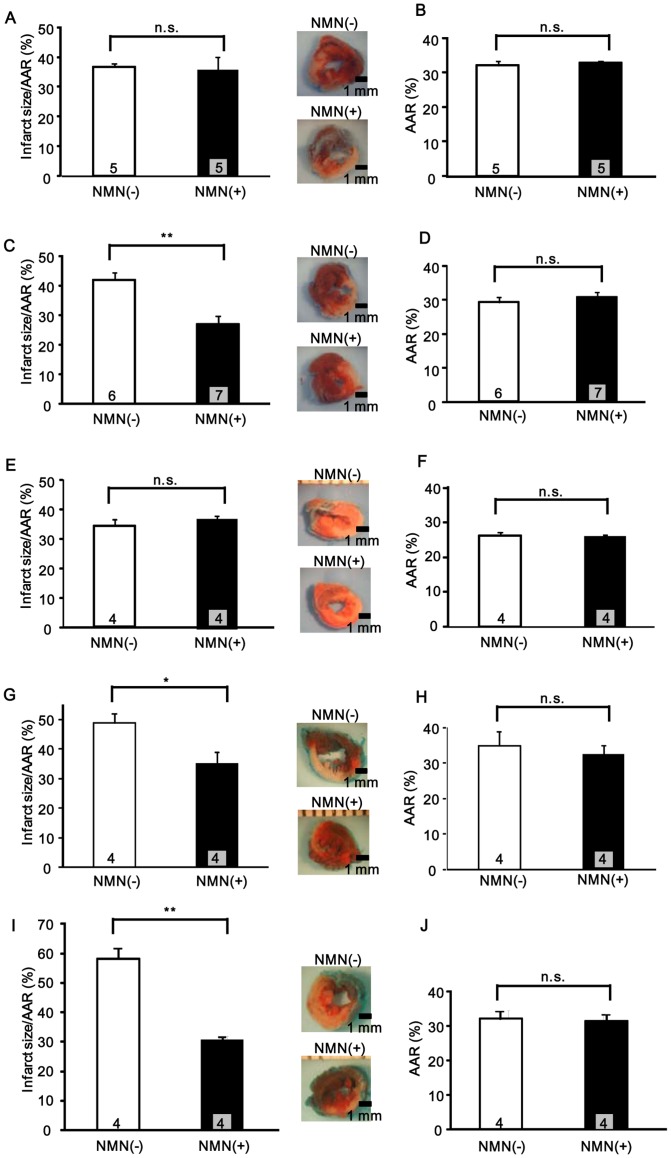
NMN administration reduces I/R injury. Either NMN (500 mg/kg per injection) or vehicle (PBS) was administered (i.p. injection) to mice according to one of four different protocols (Figure S4 in File S1), the mice were subjected to I/R, and the extent of I/R injury was evaluated with TTC staining. In A-H, control mice were used. NMN or PBS was injected once 12 hours before I/R (A and B), once 30 minutes before I/R (C and D), once just before reperfusion (E and F) or once just before reperfusion and 3 more times every 6 hours thereafter (G and H). In I and J, NMN or PBS was injected once 30 minutes before I/R in Nampt+/− mice. Infarct area/AAR (A, C, E, G, and I) and AAR (B, D, F, H, and J) are shown. n = 4 to 7. n.s., not significant; * p<0.05, ** p<0.01.

The data shown above suggest that decreases in the NAD^+^ content during myocardial ischemia can be reversed by the administration of NMN. We hypothesized that NMN may mimic the effect of IPC. To test this hypothesis, we injected NMN (500 mg/kg, i.p., once or 4 times) into mice, and its effect upon I/R injury was investigated. In order to explore the timing of the maximum effect of NMN in reducing the infarct size, NMN was applied with 4 different protocols: group 1, once 12 hours before ischemia; group 2, once 30 min before ischemia; group 3, once immediately before reperfusion; group 4, once immediately before reperfusion and 3 times thereafter at 6 hours intervals (Figure S4 in File S1). The extent of the AAR was not significantly affected by NMN in any group. While NMN administration 12 hours before ischemia and immediately before reperfusion (group 1 and group 3) did not cause any significant reduction in infarct size (group 1: AAR: NMN = 33±0.6%, vehicle = 32±1.2%, n.s.; IA/AAR: NMN = 36±4.7%, vehicle = 37±1.3%, n.s., n = 5, group 3: AAR: NMN = 26±0.5%, vehicle = 27±1.0%, n.s.; IA/AAR: NMN = 37±1.4%, vehicle = 34±2.2%, n.s., n = 4), NMN administration 30 min before ischemia and repetitive administration just before and during reperfusion (group 2 and group 4) reduced the infarct size by 44% and 29%, respectively, compared to their respective vehicle groups (group 2: AAR: NMN = 30±1.9%, vehicle = 29±1.7%, n.s.; IA/AAR: NMN = 23±2.9%, vehicle = 41±2.6%, p<0.01, n = 6 to 7, group 4: AAR: NMN = 32±2.7%, vehicle = 35±4.2%, n.s.; IA/AAR: NMN = 35±4.4%, vehicle = 49±3.2%, p<0.05, n = 4) (Figure 4A-H and Figure S5 in File S1). These results suggest that exogenous NMN has a timing-dependent ability to reduce the infarct size in response to I/R. Considering the transient action of NMN (Figure 3A), the fact that group 2, but not group 3, exhibited protection suggests that NMN action is more critical for protection during ischemia than during the early phase of reperfusion. However, the fact that group 4 also exhibited protection suggests that continuous elevation of NMN throughout reperfusion for 24 hours can also protect the heart from I/R injury. Taken together, the data suggest that exogenous application of NMN can mimic the effect of IPC.

Since NMN is produced by an enzymatic reaction of Nampt with nicotinamide and readily converted to NAD+, supplementation of NMN to mice with reduced expression of Nampt, namely Nampt +/− mice, should be protective against I/R. In fact, application of NMN to Nampt +/− mice 30 min before ischemia significantly reduced I/R injury compared to PBS alone (Figure 4I and 4J), consistent with the notion that NMN acts downstream of Nampt to induce cardioprotection.

To further confirm the protective effect of NMN applied 30 min before ischemia, we conducted echocardiographic measurements 7 days after I/R. As expected, LV systolic function was significantly decreased after I/R in both PBS and NMN treated groups compared to in mice subjected to sham operation. However, LV systolic function was significantly better after I/R in the NMN treated group than in the PBS treated group (Table S3 in File S1). This is consistent with our findings regarding the size of the myocardial infarct and confirms the protective effect of NMN against I/R injury.

### The ability of NMN to reduce infarct size depends upon Sirt1 expression

**Figure 5:**
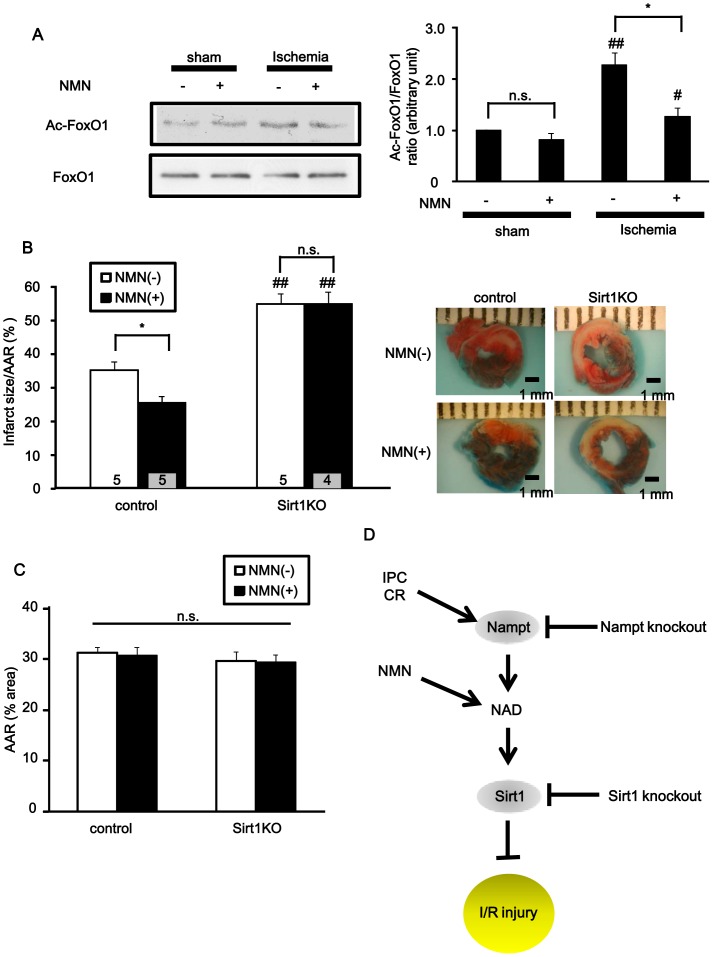
The cardioprotective effect of NMN depends on Sirt1 expression. A, Acetylation levels of FoxO1 after 30 minutes of ischemia or sham operation with or without NMN administration. n = 4. n.s., not significant; # p<0.05, ## p<0.01 vs. respective sham-operated group. B and C, Either NMN (500 mg/kg) or vehicle (PBS) was administered (i.p. injection) to control or cardiac-specific Sirt1 knockout (Sirt1KO) mice 30 minutes before subjecting the mice to 30 minutes of ischemia followed by 24 hours of reperfusion (I/R). The extent of infarction was evaluated with TTC staining. Infarct area/AAR (B) and AAR (C) are shown. n = 4 to 5. n.s., not significant; * p<0.05, ## p<0.01 vs. respective control mice group. D, Schematic model of the protective effect of NMN against I/R injury. IPC: ischemic preconditioning; CR: caloric restriction; NMN: nicotinamide mononucleotide; NAD: nicotinamide adenine dinucleotide; I/R: ischemia/reperfusion.

NAD^+^ is a coenzyme of multiple enzymatic reactions. Given that Sirt1, an NAD^+^-dependent protein deacetylase, protects the heart from I/R injury [8], the protective effect of NMN against I/R may be mediated through activation of Sirt1. Myocardial ischemia significantly increased acetylation of FoxO1, a major target of Sirt1, whereas NMN significantly attenuated ischemia-induced increases in FoxO1 acetylation (Figure 5A). Based on these results, we hypothesized that the deacetylation activity of Sirt1 may contribute to the protective effect of NMN against I/R. To test this hypothesis, cardiac-specific Sirt1 knockout (Sirt1 c-KO) and control mice were subjected to I/R with or without NMN administration 30 min before ischemia (Figure 5B and 5C). In the control mice, the infarct was significantly smaller in the NMN-administered group than in the vehicle-treated group (AAR: NMN = 30±1.7%, vehicle = 31±1.1%, n.s.; IA/AAR: NMN = 24±1.9%, vehicle = 33±2.5%, p<0.05, n = 5). The infarct was significantly larger in vehicle-administered Sirt1c-KO mice than in vehicle-administered control mice (AAR: Sirt1 c-KO = 29±1.9%, control = 31±1.1%, n.s.; IA/AAR: Sirt1 c-KO  = 51±2.9%, control = 33±2.5%, p<0.01, n = 5). Furthermore, NMN administration failed to reduce the infarct size in Sirt1c-KO mice (AAR: NMN = 29±1.5%, vehicle = 29±1.9%, n.s.; IA/AAR: NMN = 51±2.9%, vehicle = 51±3.4%, n.s., n = 4 to 5). This suggests that the protective effect of NMN against I/R injury is in part mediated through Sirt1, an NAD^+^-dependent enzyme (Figure 5D).

### The ability of caloric restriction (CR) to reduce infarct size depends upon Sirt1 expression

**Figure 6:**
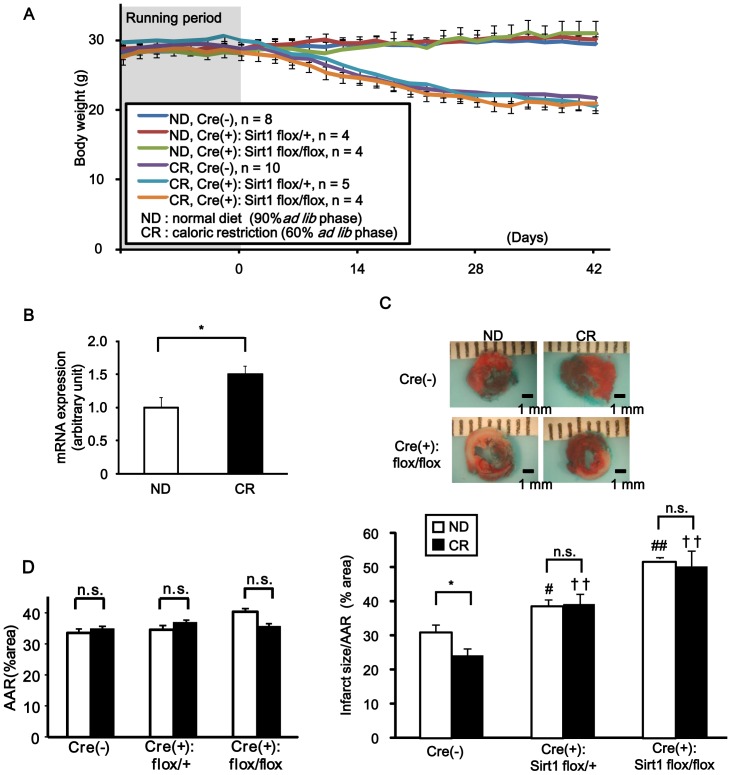
The cardioprotective effect of caloric restriction depends on Sirt1 expression. Control mice (Cre(-) Sirt1*^flox/flox^*) or cardiac-specific Sirt1 heterozygous (Cre(+): Sirt1*^flox/+^*) or homozygous (Cre(+): Sirt1*^flox/flox^*) KO mice were subjected to either caloric restriction (CR) or normal diet (ND) feeding for 6 weeks and then subjected to 30 minutes of ischemia followed by 24 hours of reperfusion (I/R). A, Time course of the body weight (g) of the mice. Before the mice were subjected to CR or ND, their eating behavior was closely observed for 14 days (running period). n = 4 to 10. B, The abundance of Nampt mRNA after 6 weeks of CR or ND was evaluated with qPCR. n = 4 to 5. * p<0.05. C and D, Infarct area/AAR (C) and AAR (D) were evaluated with injection of Alcian Blue dye and TTC staining. n = 3 to 6. n.s., not significant; * p<0.05, # p<0.05 vs. Cre(-) ND group, ## p<0.01 vs. Cre(-) ND group, †† p<0.01 vs. Cre(-) CR group.

The results presented thus far suggest that an intervention that increases the level of NAD^+^ and/or expression of Nampt and/or Sirt1 may be effective in reducing I/R injury in the heart. Accumulating lines of evidence show that CR protects the heart from I/R injury and that it is accompanied by increases in Sirt1 expression in the nucleus [17]–[19]. Whether CR affects Nampt expression remains to be elucidated. To this end, mice were fed a CR diet (60% of the caloric intake of mice fed *ad libitum*) or normal diet (ND) (90% of the caloric intake of mice fed *ad libitum*) for 6 weeks. The effectiveness of CR was confirmed by significant body weight loss in the CR group but not in the ND group (Figure 6A). The level of Nampt mRNA was significantly greater in the CR group than in the ND group (Figure 6B). As expected, the extent of myocardial infarction was significantly smaller in mice subjected to CR than in those subjected to ND (Figure 6C). In order to elucidate the role of Sirt1 in mediating CR-induced protection against I/R injury, control mice, cardiac-specific Sirt1 heterozygous KO mice (Sirt1^flox/+^- αMHC-Cre), and cardiac-specific Sirt1 homozygous KO mice (Sirt1^flox/flox^- αMHC-Cre) were fed with CR diet or ND for 6 weeks and then subjected to I/R (Figure 6C and 6D). The extent of the AAR was similar in all groups. Consistent with our previous report [8], the infarct was significantly larger in Sirt1^flox/+^- αMHC-Cre and Sirt1^flox/flox^- αMHC-Cre mice than in control mice fed with CR or ND. Furthermore, CR decreased the infarct size in the control group (AAR: ND = 33±1.4%, CR = 34±2.3%, n.s.; IA/AAR: ND = 31±2.1%, CR = 24±1.8%, p<0.05, n = 6), whereas CR did not decrease the infarct size in cardiac-specific Sirt1 heterozygous or homozygous knockout mice (Sirt1 hetero KO: AAR: ND = 34±1.7%, CR = 36±2.4%, n.s.; IA/AAR: ND = 39±1.9%, CR = 39±3.2%, n.s., n = 3 to 4; Sirt1 homo KO: AAR: ND = 40±1.3%, CR = 35±3.0%, n.s.; IA/AAR: ND = 52±0.2%, CR = 50±4.5%, n.s., n = 3). These results suggest that the protective effect of CR against I/R is mediated through Sirt1.

### Mechanisms mediating the cardioprotective effect of NMN

We investigated the molecular mechanism through which NMN mediates its cardioprotective effects against I/R. It has been shown that Nampt affects I/R injury through its effects upon neutrophil infiltration [20]. In order to evaluate whether NMN treatment affects neutrophil infiltration, we conducted immunostaining of tissue sections 24 hours after I/R, using anti-mouse Ly-6B.2 antibody. Although neutrophil infiltration was significantly increased by I/R, it was not significantly affected by NMN (Figure S6 in File S1). We also evaluated the serum levels of CXCL1 and CXCL2 [20], neutrophil chemoattractants, 1 and 24 hours after I/R. Again, NMN did not significantly affect the levels of either CXCL1 or CXCL2 (Figure S7 in File S1). These results suggest that NMN does not significantly affect neutrophil-induced injury in the mouse heart.

We also evaluated the effect of NMN upon the activity of Akt, ERK1/2 and STAT-3, well-established mechanisms of cardioprotection [1], using homogenates obtained from hearts subjected to either 30 minutes of ischemia followed by 1 hour of reperfusion or sham operation. NMN or PBS was applied 30 minutes before ischemia. Although phosphorylated STAT-3 was significantly increased by NMN compared to PBS in sham-operated hearts, it was not increased by NMN after I/R (Figure S8 in File S1). There was no significant difference in either phosphorylated Akt or phosphorylated ERK1/2 between NMN and PBS in hearts subjected to either sham operation or I/R (Figure S8 in File S1). Although the contribution of the NMN-induced upregulation of STAT3 seen in sham-operated hearts to cardioprotection by NMN against I/R injury remains to be elucidated, it is unlikely that Akt, ERK1/2 or STAT3 plays an important role in mediating the protective effect of NMN. We also investigated the effect of NMN upon protein expression of anti-oxidant and anti- and pro-apoptotic molecules, including thioredoxin1, Bcl-2, Bcl-xL and Bax (Figure S9 in File S1). NMN did not significantly affect the level of these molecules either.

**Figure 7:**
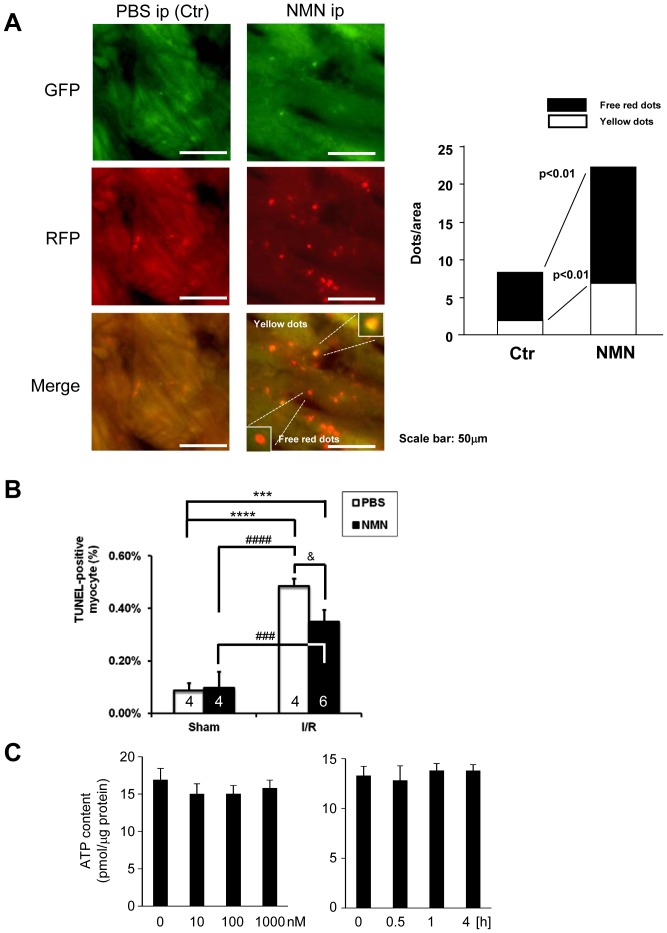
NMN stimulates autophagy in control hearts. A, Either NMN (500 mg/kg per injection) or vehicle (PBS) was administered (i.p. injection) to Tg-mRFP-GFP-LC3 mice. After 2 hours, the number of fluorescent LC3 dots was evaluated. Representative images of GFP puncta, mRFP puncta and their merged images are shown. The results of the quantitative analysis of RFP only dots and RFP/GFP double positive (yellow) dots/area are also shown. B, Either NMN (500 mg/kg per injection) or vehicle (PBS) was administered (i.p. injection) to mice 30 minutes before I/R, and then the mice were subjected to either 30 minutes ischemia followed by 24 hours of reperfusion or sham operation. The extent of cardiomyocyte apoptosis in the border zone was evaluated with TUNEL staining. The results of the quantitative analysis of TUNEL-positive cardiomyocytes are shown. C. Three days after isolation, cardiomyocytes were treated with the indicated dosage of NMN for 30 minutes (Left) and with 1000 nM NMN for the indicated time (Right). ATP contents were measured by ATP Bioluminescent Assay Kit (Sigma). n = 4 (Left) and 3(Right).

We also investigated the effect of NMN upon autophagy in the heart by treating mRFP-GFP-LC3 mice with NMN. Autophagic puncta were evaluated 2 hours after NMN treatment. Both autophagosomes, indicated by red and green puncta shown as yellow puncta in merged images, and autolysosomes, indicated by red puncta, were increased in response to NMN treatment (Figure 7A), suggesting that NMN stimulates autophagy. This is consistent with our previous observations that both Nampt and Sirt1 promote autophagy in cardiomyocytes [13], [16]. Furthermore, NMN significantly reduced the number of TUNEL- positive cardiomyocytes in the peri-infarct area after I/R (Figure 7B). NMN did not significantly affect the level of ATP in mitochondria (Figure 7C). Taken together, these results suggest that NMN may protect the heart through stimulation of autophagy during myocardial ischemia and consequent suppression of cardiomyocyte apoptosis.

## Discussion

We here demonstrated that Nampt is upregulated by IPC. Using Nampt +/− mice, we demonstrated that Nampt plays an essential role in mediating the cardioprotective effect of IPC against I/R. Furthermore, exogenous application of NMN or CR mimics the effect of IPC and overexpression of Nampt, thereby protecting the heart against I/R through a Sirt1-dependent mechanism.

### Nampt is a critical enzyme mediating NAD^+^ production in the heart in vivo

We have shown previously that Nampt is a key enzyme mediating the production of NAD^+^ in cardiomyocytes *in vitro* [13]. Using Nampt +/− mice, we show here that Nampt is also critical in maintaining the level of NAD^+^ in the adult heart *in vivo*. Overexpression of Nampt attenuates myocardial infarct size after I/R [13]. The fact that myocardial infarction after I/R is exacerbated in Nampt +/− mice suggests that *endogenous* Nampt protects the heart from I/R injury *in vivo*, consistent with our previous observation [13].

### Nampt plays an important role in mediating the effect of IPC

IPC is believed to be one of the most effective interventions for reducing I/R injury [6]. We here demonstrate that protein expression of Nampt is upregulated after 24 hours after IPC. Using Nampt +/− mice, in which IPC-induced upregulation of Nampt is abolished, we show that Nampt plays an essential role in mediating the cardioprotective effect of IPC against I/R injury. Although previous work has shown that IPC-induced protection against I/R is inhibited by FK-866, a chemical inhibitor of Nampt, in the *ex vivo* Langendorff perfused heart [7], whether Nampt is upregulated by IPC has not been shown, and the role of Nampt has not been tested with a genetically altered mouse model or in an *in vivo* model of I/R. Furthermore, our loss-of-function study shows that Nampt is involved in both the first and second windows [6] of IPC. Interestingly, protein upregulation of Nampt was not obvious 20 min after IPC, despite the fact that ischemia-induced decreases in NAD^+^ in the heart were diminished, suggesting that Nampt may undergo posttranslational modification or translocation in order to support increased production of NAD^+^. Alternatively, molecules whose expression levels are altered by chronic reduction of NAD^+^ in Nampt +/− mice may mediate the first window of preconditioning.

Although IPC upregulated Nampt, it did not significantly increase the level of NAD^+^ in the heart at baseline. We have shown previously that overexpression of Nampt in the heart increased NAD^+^ at baseline [13]. We speculate that the extent of upregulation of Nampt by IPC may not be sufficient to increase NAD^+^ under basal conditions. However, it does appear to be sufficient to prevent decreases in NAD^+^ during ischemia (Figure 2C). The NAD^+^ content may be decreased during ischemia through downregulation of Nampt. The fact that preservation of the NAD^+^ level by IPC protects the heart from I/R injury and that the protective effect of IPC is abolished in Nampt +/− mice suggest that the decrease in NAD^+^ during ischemia is detrimental and that the protective effect of IPC is mediated through preservation of NAD^+^ through upregulation of Nampt.

### NMN transiently increases NAD^+^ content in the heart

Since Nampt is a rate-limiting enzyme in NAD^+^ synthesis in the heart [13], we hypothesized that NMN, the product of the enzymatic reaction of Nampt, can increase NAD^+^ content in the heart and mimic the protective effect of Nampt overexpression against I/R injury. We chose the administration of NMN, rather than NAD^+^, because NAD^+^ is less soluble in PBS than NMN and is taken up or penetrates less efficiently through the plasma membrane [21]. We showed that i.p. injection of NMN successfully increases NAD^+^ content in the heart within 30 min, although the increase in NAD^+^ content in the heart decreased time-dependently. Importantly, i.p. application of NMN 30 min before ischemia prevented the ischemia-induced decreases in NAD^+^ content in the heart, suggesting that i.p. application of NMN is able to supplement the amount of NAD^+^ in the heart during ischemia.

The fact that application of NMN either once 30 min before ischemia or 4 times every 6 hours during reperfusion significantly reduced I/R injury suggests that increases in NAD^+^ during both ischemia and reperfusion effectively prevent I/R injury. The fact that the application of NMN once 30 min before ischemia was effective, whereas once just before reperfusion was not, clearly suggests that NAD^+^ has protective effects during ischemia. Although it is widely believed that myocardial injury occurs very early during reperfusion [1], application of NMN only once just before reperfusion was not sufficient to reduce the myocardial infarct size. Interestingly, however, multiple applications of NMN during reperfusion were effective in reducing myocardial injury. Thus, if this intervention is considered for clinical application, caution should exercised to maintain the level of NMN/NAD+ for a sufficiently long period during reperfusion in order to reduce I/R injury. We have shown previously that cardiac-specific overexpression of Nampt in mice protects the heart from both prolonged ischemia and I/R [13]. The results obtained with NMN not only support the results obtained with Tg-Nampt but also suggest that the effect of NAD^+^ is rapid and may not require long-term changes in gene expression or cardiac structure, such as angiogenesis. Although Nampt can be secreted into the extracellular space and act as a cytokine [22], the ability of NMN to mimic the protection observed in Tg-Nampt suggests that the effect of Nampt overexpression is mediated through its action as an enzyme stimulating the synthesis of NAD^+^.

### The effect of NMN is mediated primarily through Sirt1

NAD^+^ is a coenzyme of the deacetylation reactions carried out by sirtuins [23]. We have shown previously that the protective effect of Nampt in cardiomyocytes is mediated through Sirt1 [13]. We here show that the acetylation level of FoxO1, an important target of Sirt1 in mediating cardioprotection [8], was increased during ischemia and was partially restored by NMN administration. Furthermore, NMN application failed to exert any protective effects in cardiac-specific Sirt1 KO mice. These results suggest that Sirt1 plays an important role in mediating the effect of the NMN treatment. Considering the fact that NAD^+^ also acts as a co-factor and/or substrate of other enzymes, such as PARP, and as an acceptor of electrons in the glycolytic pathway and the TCA cycle [24], it is remarkable that exogenous NAD^+^ is effectively used by Sirt1 for cardioprotection. This suggests that the salvage pathway of NAD^+^ synthesis effectively couples with Sirt1 and that Sirt1 plays a major role in mediating protection against I/R.

### Interventions to upregulate Nampt

Although NMN protects the heart from I/R injury, its instability in the systemic circulation makes it impractical for clinical use for induction of pharmacological preconditioning. We here show that CR, which protects the heart from I/R injury, induces upregulation of Nampt. Since CR failed to induce cardioprotection in Sirt1 KO mice, it is likely that the cardioprotective effect of CR is in part mediated through activation of the Nampt-Sirt1 pathway.

The molecular mechanism regulating Nampt expression during IPC and CR remains to be elucidated. Nampt is regulated by HIF-1α [25], FoxO [26], and LXR [27] transcription factors, as well as through an AMPK-dependent mechanism [28]. Expression of Nampt is also regulated by CLOCK:BMAL1, the key genes in the circadian mechanism [29]. However, the involvement of these various mechanisms in the upregulation of Nampt during IPC remains to be determined. We have shown previously that Nampt is downregulated by I/R and prolonged ischemia [13]. Thus, signaling mechanisms uniquely activated by IPC, but not those activated by I/R or prolonged ischemia, appear to mediate the upregulation of Nampt. Elucidating the molecular mechanism by which Nampt expression is regulated may allow us to develop alternative interventions for protection against I/R injury through upregulation of Nampt.

### Limitations of this study

The cardioprotective effect of NMN during ischemia and reperfusion requires more investigation. Thus far, we have found that autophagic flux is stimulated by NMN. However, it remains unknown whether this promotes survival of cardiomyocytes during both ischemia and reperfusion. Furthermore, the effect of NMN appears to be largely mediated through Sirt1 but whether the cardioprotective effect of NMN is predominantly mediated through Sirt1-induced activation of autophagy remains to be shown. We used CR as a measure to induce upregulation of Nampt. However, the mechanism of CR is complex. The role of other signaling mechanisms, including AMPK activation and mTOR suppression, in mediating protection by CR remains to be elucidated. Finally, some I/R experiments were conducted with a relatively small number of mice due to limited availability of mice and, thus, they await confirmation by further experimentation.

## Supporting Information

File S1 **Supporting tables and figures.** Table S1. Cardiac function in Nampt+/- mice at baseline Table S2. Organ weights in Nampt+/− mice at baseline Table S3. Cardiac function in wild-type mice undergone I/R with or without NMN administration Figure S1. Nampt protein abundance in the various organs of Nampt +/− mice. Figure S2. The protocol used for I/R with or without IPC in Figure 1E-G. There are three groups. In the first group, mice were subjected to I/R without IPC. In the second group, mice were subjected to I/R 5 minutes after IPC. In the third group, mice were subjected to I/R 24 hours after IPC. In all groups, TTC staining was conducted 24 hours after I/R. Figure S3. The protocol used for 30 minutes ischemia with or without IPC in Figure 2. Mice were subjected to IPC or sham procedure and then subjected to 30 minutes of ischemia either 5 minutes or 24 hours after IPC. Figure S4. The protocol used for I/R with or without NMN in Figure 4. Either NMN (500 mg/kg per injection) or vehicle (PBS) was administered (i.p. injection) to mice according to one of four different protocols, the mice were subjected to I/R, and the extent of I/R injury was evaluated with TTC staining. NMN or PBS was injected once 12 hours before I/R, once 30 minutes before I/R, once just before reperfusion or once just before reperfusion and 3 more times every 6 hours thereafter. Figure S5. Large magnification of TTC staining shown in Figure 4. The area of myocardial infarction is demarcated by black lines. Figure S6. The effect of NMN upon neutrophil infiltration. Either NMN (500 mg/kg per injection) or vehicle (PBS) was administered (i.p. injection) to mice once 30 minutes before I/R and then mice were subjected to I/R or sham operation. Twenty-four hours after I/R, the heart was fixed with formalin and immunostained with anti-Ly-6B.2 antibody. The upper panel shows representative staining and the lower panel shows quantitative analyses. NMN did not significantly affect neutrophil infiltration. n = 4–5. Figure S7. The effect of NMN upon the serum levels of CXCL1 and CXCL2. Either NMN (500 mg/kg per injection) or vehicle (PBS) was administered (i.p. injection) to mice 3 once 30 minutes before I/R and then mice were subjected to I/R or sham operation. One and 24 hours after reperfusion, serum levels of CXCL1 and CXCL2 were evaluated with ELISA. n = 4. Figure S8. Either NMN (500 mg/kg per injection) or vehicle (PBS) was administered (i.p. injection) to mice once 30 minutes before I/R and then mice were subjected to I/R or sham operation. One hour after I/R, the heart was harvested. The heart homogenates were prepared and Western blot analyses were performed with the indicated antibodies. Figure S9. Either NMN (500 mg/kg per injection) or vehicle (PBS) was administered (i.p. injection) to mice once 30 minutes before I/R and then mice were subjected to I/R or sham operation. One hour after I/R, the heart was harvested. The heart homogenates were prepared and Western blot analyses were performed with the indicated antibodies.
(PDF)
