# Supplementary material for: Nicotinamide Mononucleotide, an Intermediate of NAD+ Synthesis, Protects the Heart from Ischemia and Reperfusion
Source: PLoS One. 2014 Jun 6;9(6):e98972. doi: 10.1371/journal.pone.0098972 (PMC4048236; doi:10.1371/journal.pone.0098972)
Supplement: File S1 — Supporting tables and figures. Table S1. Cardiac function in Nampt+/- mice at baseline Table S2. Organ weights in Nampt+/− mice at baseline Table S3. Cardiac function in wild-type mice undergone I/R with or without NMN administration Figure S1. Nampt protein abundance in the various organs of Nampt +/− mice. Figure S2. The protocol used for I/R with or without IPC in Figure 1E-G. There are three groups. In the first group, mice were subjected to I/R without IPC. In the second group, mice were subjected to I/R 5 minutes after IPC. In the third group, mice were subjected to I/R 24 hours after IPC. In all groups, TTC staining was conducted 24 hours after I/R. Figure S3. The protocol used for 30 minutes ischemia with or without IPC in Figure 2. Mice were subjected to IPC or sham procedure and then subjected to 30 minutes of ischemia either 5 minutes or 24 hours after IPC. Figure S4. The protocol used for I/R with or without NMN in Figure 4. Either NMN (500 mg/kg per injection) or vehicle (PBS) was administered (i.p. injection) to mice according to one of four different protocols, the mice were subjected to I/R, and the extent of I/R injury was evaluated with TTC staining. NMN or PBS was injected once 12 hours before I/R, once 30 minutes before I/R, once just before reperfusion or once just before reperfusion and 3 more times every 6 hours thereafter. Figure S5. Large magnification of TTC staining shown in Figure 4. The area of myocardial infarction is demarcated by black lines. Figure S6. The effect of NMN upon neutrophil infiltration. Either NMN (500 mg/kg per injection) or vehicle (PBS) was administered (i.p. injection) to mice once 30 minutes before I/R and then mice were subjected to I/R or sham operation. Twenty-four hours after I/R, the heart was fixed with formalin and immunostained with anti-Ly-6B.2 antibody. The upper panel shows representative staining and the lower panel shows quantitative analyses. NMN did not significantly affect neutrophil infiltration. n [file pone.0098972.s001.pdf]

Table S1. Cardiac function in Nampt+/- mice at baseline

	Wt (n = 7)	Nampt+/- (n = 6)
HR (bpm)	406 ± 25	421± 25
IVSt (mm)	0.80 ± 0.02	0.77 ± 0.02
PWt (mm)	0.83 ± 0.02	0.78 ± 0.03
LVDd (mm)	3.34 ± 0.15	3.39 ± 0.14
LVDs (mm)	2.21 ± 0.13	2.23 ± 0.11
EF (%)	70.8 ± 2.4	71.6 ± 1.5

IVSt, intraventicular septal wall thickness; PWt, posterior wall thickness; LVDd, end-diastolic dimension of LV; LVDs, end-systolic dimension of LV; EF, ejection fraction. Each mouse is male 3 to 4 months old. No significant difference in each value between 2 groups.

Table S2. Organ weights in Nampt+/- mice at baseline

	Wt (n = 6)	Nampt+/- (n = 6)
HW/TL (mg/mm)	6.93 ±0.26	6.74 ±0.10
LA/TL (mg/mm)	0.23 ± 0.02	0.22 ± 0.01
RA/TL (mg/mm)	0.18 ± 0.01	0.18 ± 0.02
LV/TL (mg/mm)	5.49 ± 0.19	5.31 ± 0.07
RV/TL (mg/mm)	1.04 ± 0.07	1.03 ± 0.04
Lung weight/TL (mg/mm)	8.50 ± 0.15	8.78 ± 0.10
Liver weight/TL (mg/mm)	73.86 ±3.52	77.51 ±2.98

HW, heart weight; TL, tibial length; LA, left atrium; RA, right atrium; LV, left ventricle; RV, right ventricle. Each mouse is male and 3 to 4 months old. No significant difference in each value between 2 groups.

Table S3. Cardiac function in wild-type mice undergone I/R with or without NMN administration

	sham NMN(-) (n = 5)	sham NMN(+) (n = 5)	I/R NMN(-) (n = 5)	I/R NMN(+) (n = 4)
HR (bpm)	412 ± 24	375 ±13	391 ± 11	402 ± 15
IVSt (mm)	0.82 ± 0.03	0.73 ± 0.02	0.73 ± 0.02	0.69 ± 0.02
PWt (mm)	0.78 ± 0.02	0.78 ± 0.04	0.82 ± 0.03	0.66 ± 0.02
LVDd (mm)	3.45 ± 0.17	3.75 ± 0.05	3.65 ± 0.31	3.89 ± 0.22
LVDs (mm)	2.02 ± 0.17	2.26 ± 0.15	2.95 ± 0.26 *	2.73 ± 0.19
EF (%)	80.0 ± 2.3	77.0 ± 4.4	45.2 ± 7.2 **	64.7 ± 4.0 #

I/R, ischemia/ reperfusion; IVSt, intraventicular septal wall thickness; PWt, posterior wall thickness; LVDd, end-diastolic dimension of LV; LVDs, end-systolic dimension of LV; EF, ejection fraction. * $p < 0.05$, ** $p < 0.01$ vs. sham NMN(-) group, # $p < 0.05$ vs. I/R NMN(+) group.

Figure S1

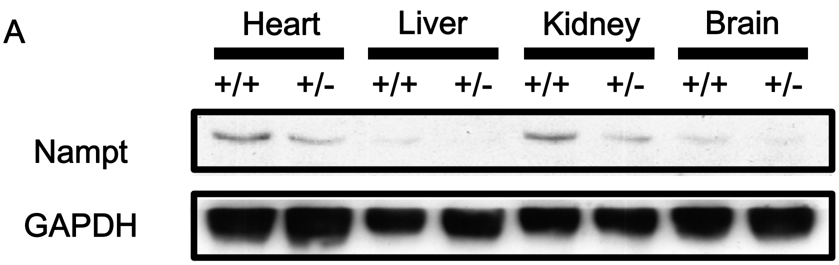

Figure S1. Nampt protein abundance in the various organs of Nampt +/- mice.

Figure S2

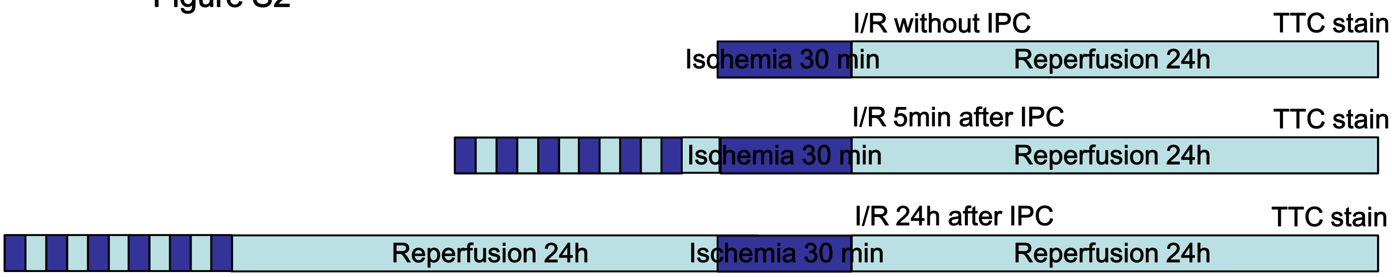

Figure S2. The protocol used for I/R with or without IPC in Figure 1E-G. There are three groups. In the first group, mice were subjected to I/R without IPC. In the second group, mice were subjected to I/R 5 minutes after IPC. In the third group, mice were subjected to I/R 24 hours after IPC. In all groups, TTC staining was conducted 24 hours after I/R.

Figure S3

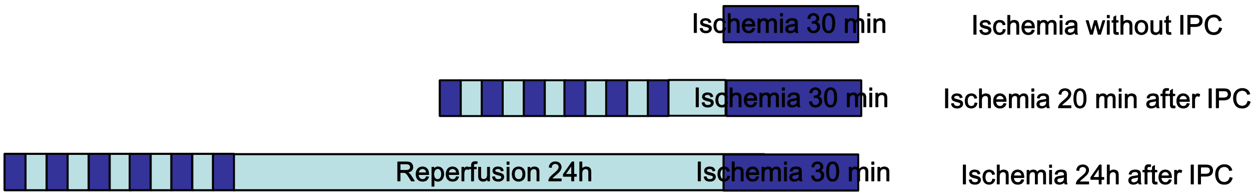

Figure S3. The protocol used for 30 minutes ischemia with or without IPC in Figure 2. Mice were subjected to IPC or sham procedure and then subjected to 30 minutes of ischemia either 5 minutes or 24 hours after IPC.

Figure S4

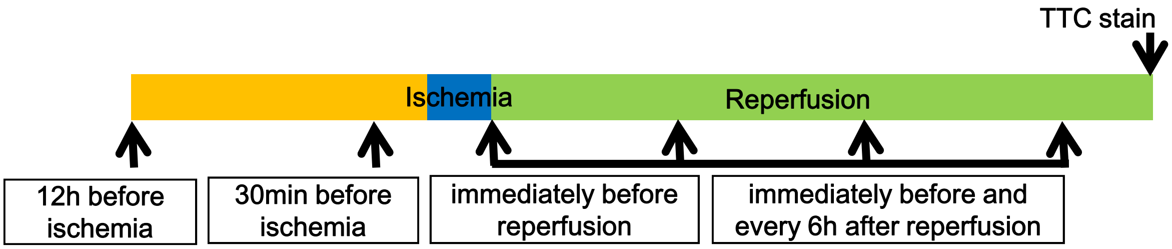

Figure S4. The protocol used for I/R with or without NMN in Figure 4.
Either NMN (500 mg/kg per injection) or vehicle (PBS) was administered (i.p. injection) to mice according to one of four different protocols, the mice were subjected to I/R, and the extent of I/R injury was evaluated with TTC staining. NMN or PBS was injected once 12 hours before I/R, once 30 minutes before I/R, once just before reperfusion or once just before reperfusion and 3 more times every 6 hours thereafter.

Figure S5

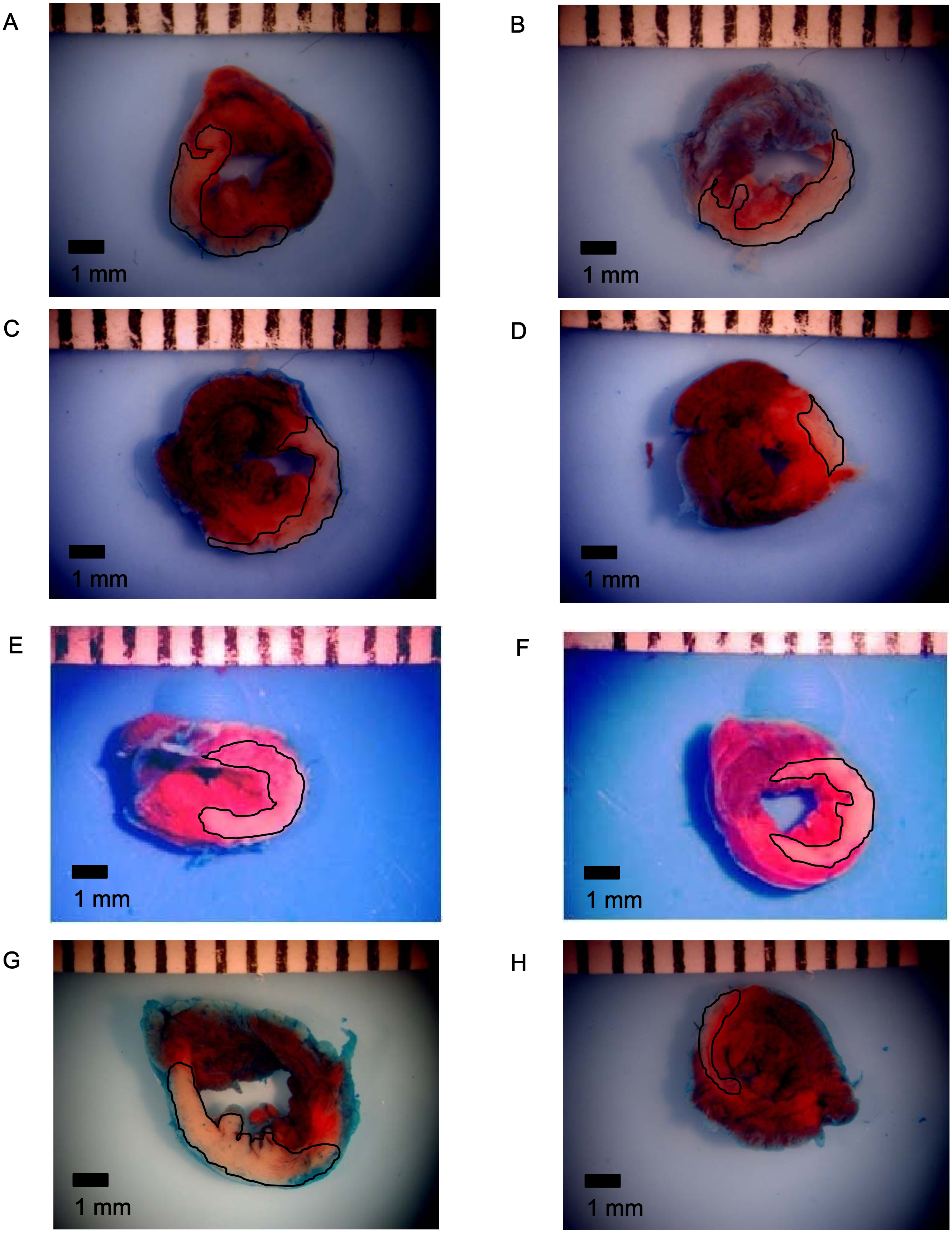

Figure S5

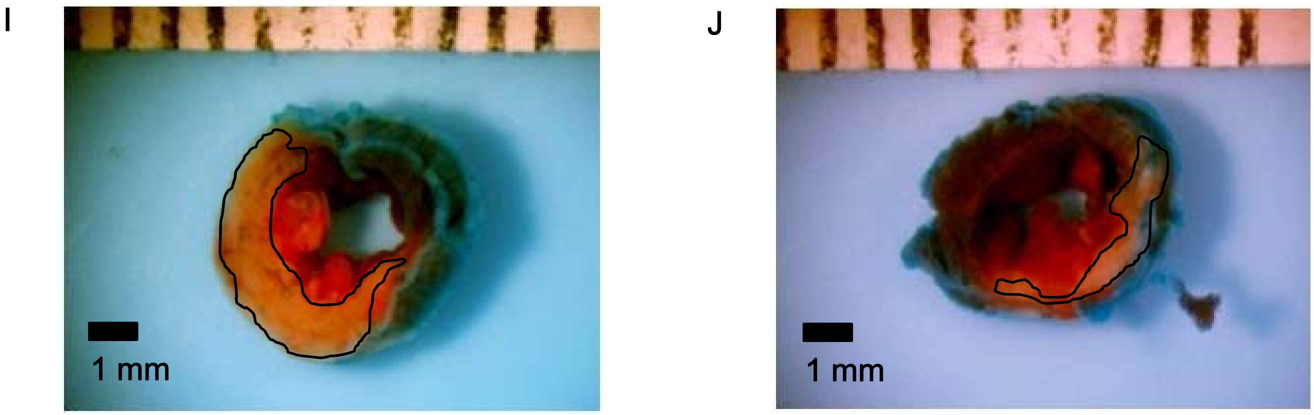

Figure S5. Large magnification of TTC staining shown in Figure 4. The area of myocardial infarction is demarcated by black lines.

Figure S6

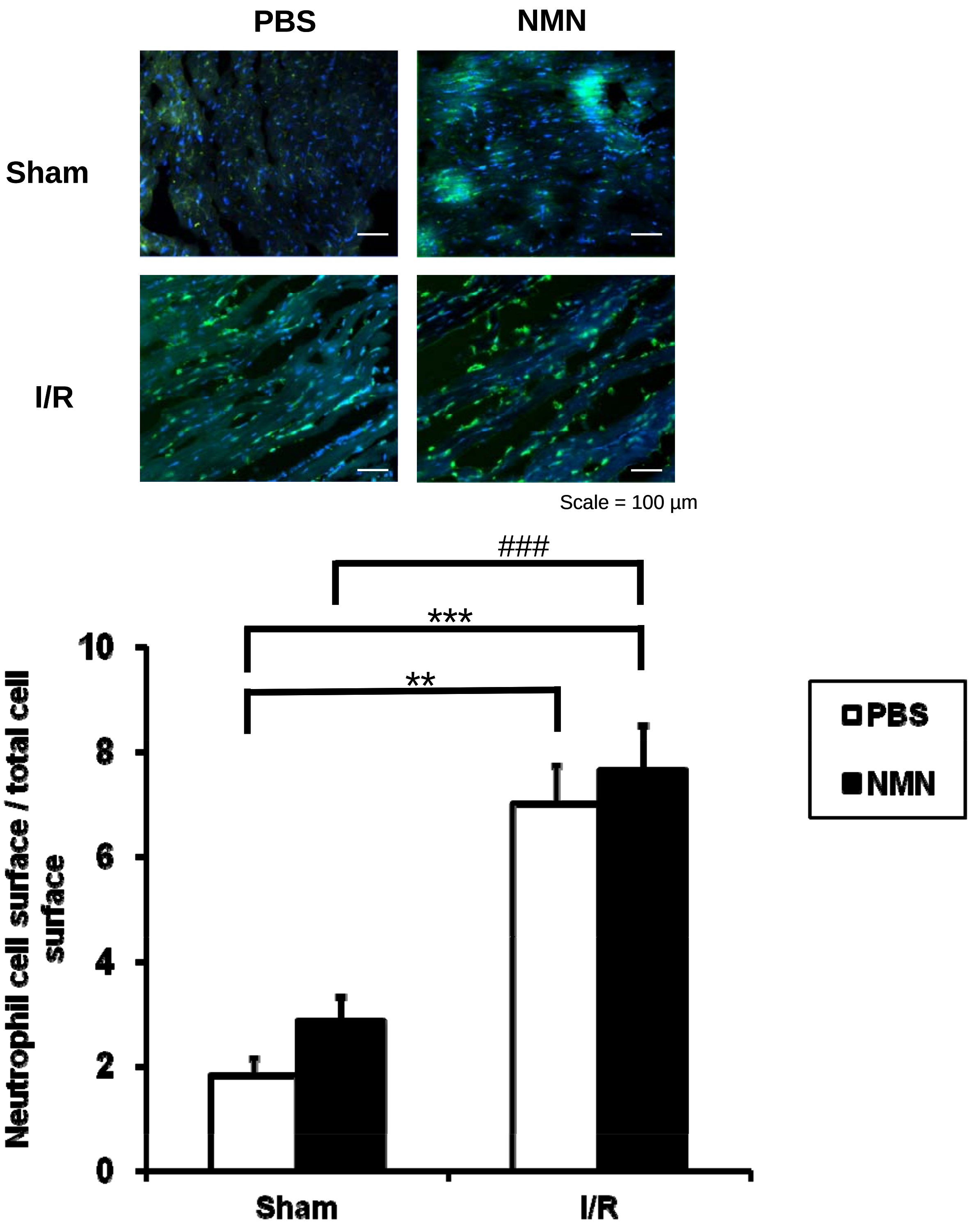

Figure S6. The effect of NMN upon neutrophil infiltration. Either NMN (500 mg/kg per injection) or vehicle (PBS) was administered (i.p. injection) to mice once 30 minutes before I/R and then mice were subjected to I/R or sham operation. Twenty-four hours after I/R, the heart was fixed with formalin and immunostained with anti-Ly-6B.2 antibody. The upper panel shows representative staining and the lower panel shows quantitative analyses. NMN did not significantly affect neutrophil infiltration. n=4-5.

Figure S7

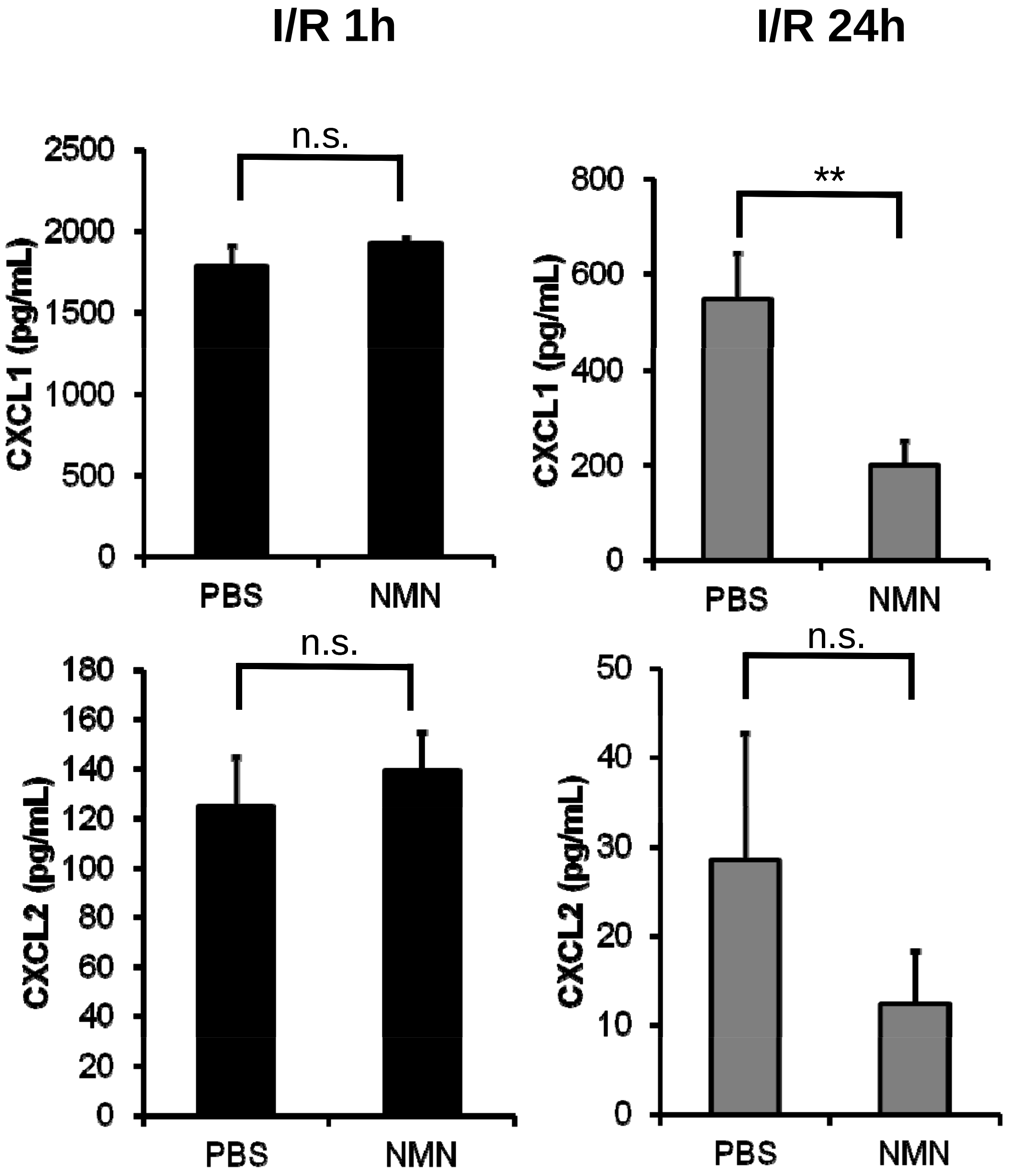

Figure S7. The effect of NMN upon the serum levels of CXCL1 and CXCL2. Either NMN (500 mg/kg per injection) or vehicle (PBS) was administered (i.p. injection) to mice 3 once 30 minutes before I/R and then mice were subjected to I/R or sham operation. One and 24 hours after reperfusion, serum levels of CXCL1 and CXCL2 were evaluated with ELISA. n=4.

Figure S8

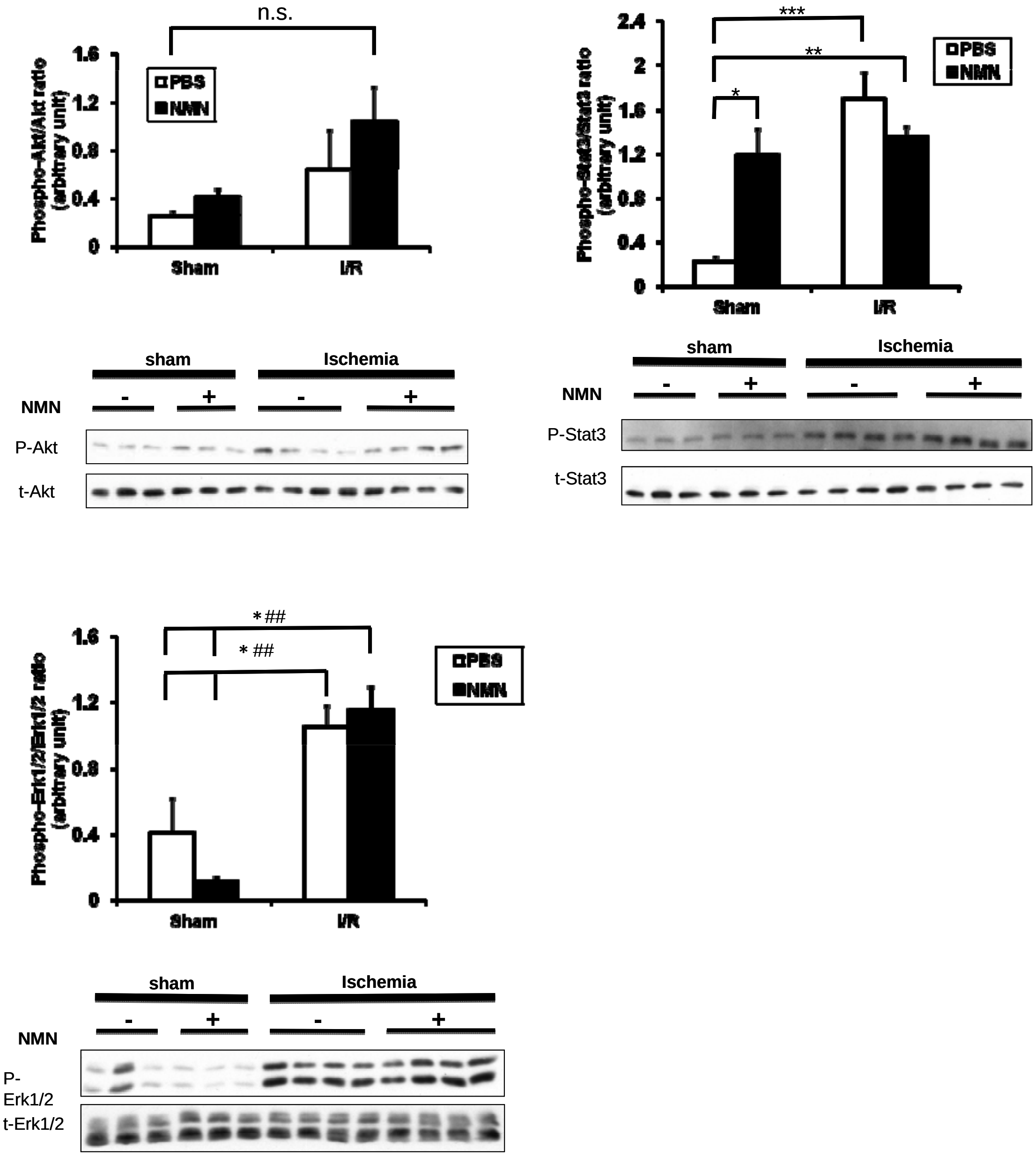

Figure S8. Either NMN (500 mg/kg per injection) or vehicle (PBS) was administered (i.p. injection) to mice once 30 minutes before I/R and then mice were subjected to I/R or sham operation. One hour after I/R, the heart was harvested. The heart homogenates were prepared and Western blot analyses were performed with the indicated antibodies.

Figure S9

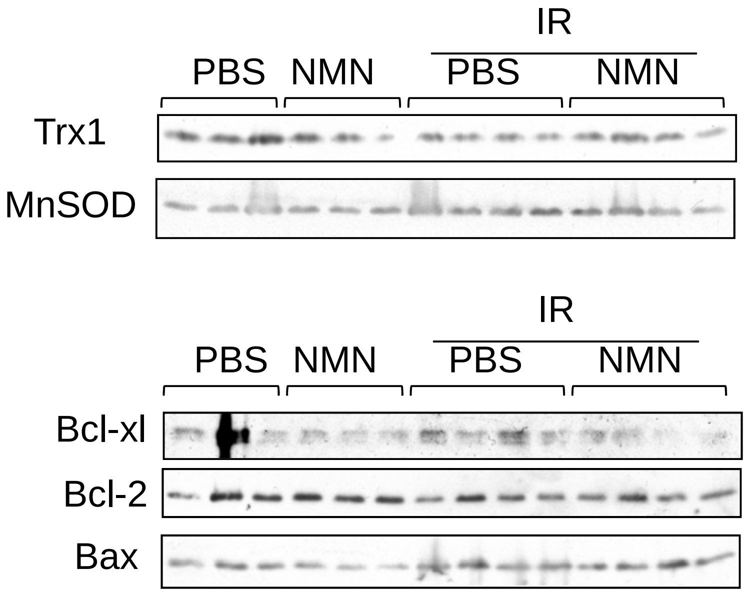

Figure S9. Either NMN (500 mg/kg per injection) or vehicle (PBS) was administered (i.p. injection) to mice once 30 minutes before I/R and then mice were subjected to I/R or sham operation. One hour after I/R, the heart was harvested. The heart homogenates were prepared and Western blot analyses were performed with the indicated antibodies.
